# Pemphigus Foliaceus Autoantibodies Induce Redistribution Primarily of Extradesmosomal Desmoglein 1 in the Cell Membrane

**DOI:** 10.3389/fimmu.2022.882116

**Published:** 2022-05-12

**Authors:** Matthias Hiermaier, Daniela Kugelmann, Mariya Y. Radeva, Dario Didona, Kamran Ghoreschi, Solimani Farzan, Michael Hertl, Jens Waschke

**Affiliations:** ^1^ Chair of Vegetative Anatomy, Institute of Anatomy, Faculty of Medicine, LMU Munich, München, Germany; ^2^ Department of Dermatology and Allergology, Philipps University, Marburg, Germany; ^3^ Department of Dermatology, Venereology and Allergology, Charité-Universitätsmedizin Berlin, Berlin, Germany; ^4^ Department of Dermatology, University Medical Center, Eberhard Karls Universität Tübingen, Tübingen, Germany

**Keywords:** pemphigus, signaling / signaling pathways, localization, desmoglein (Dsg), Stimulated Emission Depletion (STED)

## Abstract

The autoimmune dermatosis pemphigus foliaceus (PF) is predominantly caused by IgG autoantibodies against the desmosomal cadherin desmoglein (Dsg) 1. The exact mechanisms that lead to the characteristic epidermal blistering are not yet fully understood. In the present study, we used a variety of biophysical methods to examine the fate of membrane-bound Dsg1 after incubation with PF patients’ IgG. Dispase-based dissociation assays confirmed that PF-IgG used for this study reduced intercellular adhesion in a manner dependent on phospholipase C (PLC)/Ca^2+^ and extracellular signal-regulated kinase (ERK) 1/2 signaling. Atomic force microscopy (AFM) revealed that Dsg1 binding on single molecule level paralleled effects on keratinocyte adhesion under the different conditions. Stimulated emission depletion (STED) super-resolution microscopy was used to investigate the localization of Dsg1 after PF-IgG incubation for 24 h. Under control conditions, Dsg1 was found to be in part co-localized with desmoplakin and thus inside of desmosomes as well as extra-desmosomal along the cell border. Incubation with PF-IgG reduced the extra-desmosomal Dsg1 fraction. In line with this, fluorescence recovery after photobleaching (FRAP) experiments demonstrated a strongly reduced mobility of Dsg1 in the cell membrane after PF-IgG treatment indicating remaining Dsg1 molecules were primarily located inside desmosomes. Mechanistically, experiments confirmed the involvement of PLC/Ca^2+^ since inhibition of PLC or 1,4,5-trisphosphate (IP3) receptor to reduce cytosolic Ca^2+^ reverted the effects of PF-IgG on Dsg1 intra-membrane mobility and localization. Taken together, our findings suggest that during the first 24 h PF-IgG induce redistribution predominantly of membrane-bound extradesmosomal Dsg1 in a PLC/Ca^2+^ dependent manner whereas Dsg1-containing desmosomes remain.

## Introduction

Desmosomes are complex cell-cell contacts mediating strong intercellular adhesion and can be found in all epithelia, especially in tissue subjected to high mechanical stress like the skin or myocardium (1, 2). They consist of desmosomal cadherins called desmoglein (Dsg) 1-4 and desmocolin (Dsc) 1-3 which are linked *via* armadillo proteins such as plakoglobin (Pg) and plakophilins to desmoplakin (Dp) that couples the protein complex to the intermediate filament network (3). Dsgs and Dscs are engaging in homo- and heterophilic trans-interactions in a Ca^2+^ dependent manner to join the intermediate filament networks of two adjacent cells together (4, 5). Although providing mechanical resilience, desmosomes are highly dynamic structures undergoing constant remodeling. Desmosomal cadherins can also be located extra-desmosomal in the cell membrane, which can serve as a pool for shuttling adhesion molecules into or out of desmosomes (6, 7).

Pemphigus is an autoimmune dermatosis characterized by epidermal blister formation and/or mucosal erosions. Genetic factors can increase the susceptibility for pemphigus and influence the intricate pathomechanisms (8, 9). While several antibodies can be found in patients’ sera, the major target antigens in pemphigus are the desmosomal cadherins Dsg1 and Dsg3 (10–12). The antibody profile correlates with the clinical phenotype of the two main variants of pemphigus. Pemphigus foliaceus (PF) patients show mainly antibodies against Dsg1 and blisters in epidermal skin, while mucocutaneous pemphigus vulgaris (PV) is characterized by additional antibodies targeting Dsg3 and blistering of the skin and mucosal erosions (13). It has been shown that fractions of pemphigus patients’ sera targeting only Dsg3 are sufficient to induce loss of keratinocyte adhesion between the basal and spinous layer of the epidermis (acantholysis) in neonatal mice, a hallmark of pemphigus (14). In contrast, in human skin, antibodies against Dsg1 are required to induce acantholysis similar to the situation in patients, indicating that Dsg1 plays an important role for maintenance of epidermal integrity (15). This is supported by animal models where loss of Dsg1 caused lethal skin blisters in superficial epidermis, whose localization is similar to patients with PF (16, 17). The mechanisms of antibody induced acantholysis are manifold and include direct inhibition of desmosomal cadherin binding as well as an activation of numerous signaling pathways such as p38MAPK, PKC, EGFR, PLC and ERK1/2 (18–20). In this regard, desmosomes are suggested to play a key role in serving as signaling hubs (18, 19, 21). Therefore, it was proposed that Dsg1 and Dsg3 mediate autoantibody-specific signaling which may contribute to the different clinical phenotypes in pemphigus (22).

It has previously been shown that Ca^2+^ signaling plays a key role in PF-IgG induced Dsg1 relocalization and loss of intercellular adhesion (23). However, little is known about Dsg1 intra-membrane dynamics or the adhesion alterations on the single molecule level in this context. In this study, we aim to enlighten the understanding of pemphigus pathogenesis by investigating the fate of Dsg1 in response to PF-IgG regarding its dynamics and intramembrane localization and the involvement of the cytosolic Ca^2+^ signaling.

## Materials and Methods

### Cell Culture

Human keratinocyte cells (HaCaT) (24) were cultured according to standard protocols in Dulbecco’s modified Eagle’s medium (DMEM) (Thermo Fisher Scientific, Waltham, MA) containing 1.8 mM Ca^2+^, supplemented with 10 % fetal bovine serum (Sigma Aldrich, St. Louis, MO ), 50 µg/ml streptomycin (AppliChem, Darmstadt, Germany), 50 U/ml penicillin (AppliChem) at 37 °C in a 5 % CO_2_ atmosphere.

### Mediators and Concentrations

The inositol 1,4,5-trisphosphate (IP3) receptor-mediated Ca^2+^ release inhibitor xestospongin C (Xesto) (Merck, Darmstadt, Germany) was used at a final concentration of 0.4 µM. To suppress PLC mediated signaling U-73122 (Merck) was used at 4 µM. The MEK1/2 inhibitor U0126 (Cell Signaling Technology, Danvers, MA) was used at 10 µM to suppress ERK1/2 activation. Since all mediators were dissolved in DMSO (Merck), control experiments were conducted in 0.2 % DMSO to match the DMSO concentration caused by treatment with mediators.

### Pemphigus Sera and IgG Purification

In this study, sera from two patients were used, one suffering from PF and one from PV, respectively. The diagnose was based on ELISA (Euroimmun, Lübeck Germany), direct immunofluorescence and histology. Clinical background information can be found in **Table 1**. Patient sera were obtained from the dermatology department of the Philipps University Marburg. Donors gave written consent for research use and a positive vote of the ethics committee from the medical faculty of the University of Marburg was given (Az20/14). Protein A affinity purification was used to obtain IgG fractions from sera of PF and PV patients as well as healthy volunteers as control-IgG (c-IgG) as described previously (25). All IgG were incubated 1:50 in the experiments which resulted in a final concentration of 29 µg/ml c-IgG, 62 µg/ml PF-IgG and 68 µg/ml PV-IgG, respectively, as evaluated using a BCA protein assay kit (Thermo Fisher Scientific) according to the manufacturers protocol. The monoclonal anti-Dsg3 antibody AK23 (Biozol, Eching, Germany) was used at 75 µg/ml.

**Table 1:** Clinical background of patient sera.

IgG	ELISA score [U/ml]		Patient age	Disease status	Therapy
	anti-Dsg1 IgG	anti-Dsg3 IgG			
PF-IgG	756	0	82	Chronic	none
PV-IgG	253	128	43	Acute relapse	Dapson 50mg

### Dispase-Based Dissociation Assay

HaCaT cells were grown in 24-well plates. After reaching confluency they were pre-incubated for 1 h with respective mediators, then IgG fractions were added for 24 h. Afterwards, cells were washed with Hank’s balanced salt solution and incubated with dispase II solution (>2.4 U/ml dispase II in Hank’s balanced salt solution, Sigma-Aldrich) for 20 min at 37 °C until the cell monolayer detached from the well bottom. An electric pipette (Eppendorf, Hamburg, Germany) was used to apply a defined mechanical stress by pipetting the monolayer 10 times. The resulting fragmentation of the cell layer, which is an inverse measurement for intercellular adhesion, was automatically analyzed using a self-written ImageJ (National Institutes of Health, Bethesda, MD) macro counting all cell fragments larger than 0.0125 mm². The number of fragments in each experimental condition has been normalized to the DMSO c-IgG value.

### Fluorescence Recovery After Photobleaching (FRAP)

FRAP studies were performed as described previously (26). Briefly, cells were seeded in eight-well imaging chambers (Ibidi, Martinsried, Germany) and transfected with the plasmid mCherry-Desmoglein1-N-18, a gift from Michael Davidson (Addgene plasmid # 55030; http://n2t.net/addgene:55030; RRID:Addgene_55030) using Turbofect (Thermo Fisher Scientific) according to the manufacturers protocol. 24 h after transfection cells were incubated with respective mediators and IgG fractions for 24 h. FRAP experiments were conducted on an SP5 inverted microscope with a x63 HC PL APO NA=1.2 objective (Leica, Wetzlar, Germany) in a cage incubator (OKOLAB, Burlingame, CA) at 37 °C at constant humidity with 5 % CO_2_. The FRAP wizard software (Leica) was used to perform and analyze the experiments. Bleaching areas were chosen along the membrane of two transfected neighboring cells. 5 frames were captured to obtain the pre bleach mCherry intensity, followed by bleaching for 10 frames using the 594 nm laser line at 100 % transmission. Recovery of the fluorescence was recorded for 180 s until a stable fluorescence intensity was reached. The fraction of immobile molecules was calculated as


$$immobile\;fraction\;=\;1-\frac{I_{plateau}-I_{bleach}}{1-I_{bleach}}$$

with *I_plateau_* being the plateau fluorescence intensity after recovery and *I*_bleach_ being the fluorescence intensity after the bleaching step, both normalized to the initial fluorescence intensity.

### Atomic Force Microscopy (AFM)

For AFM experiments, an atomic force microscope (Nanowizard IV, JPK Instruments, Berlin, Germany) mounted on an inverted microscope (IX83, Olympus, Tokyo, Japan) was used. AFM cantilevers (MLCT AFM Probes, Bruker, Calle Tecate, CA) were functionalized with recombinant Dsg1-FC as described previously (26, 27). AFM experiments were conducted on living HaCaT cells at 37 °C in DMEM medium containing 1.8 mM Ca^2+^. After acquisition of a topographic image to identify cell borders, sampling areas of 2 µm x 2 µm over the nucleus and 5 µm x 1.5 µm across cell borders between two cells were selected and force-distance-curves (FDC) were recorded in a pixel-wise manner. For each FDC, the cantilever tip was brought into contact with the cells for 0.1 s with an indentation force of 0.5 nN and then retracted 3 µm at 10 µm/s. The resulting FDCs were analyzed using the JPKSPM Data Processing software (version 6, JPK Instruments) and for each unbinding event the corresponding unbinding force was determined. For each experiment, an interaction probability was calculated as the ratio of FDCs that showed an interaction and the total number of FDCs. For each experiment, the most probable unbinding force was computed by fitting the distribution of the forces with an extreme function of the form $y=y_{0}+A\;\exp\;\left[-\exp\left[-\left(\frac{x-x_{c}}{w}\right)\right]-\left(\frac{x-x_{c}}{w}\right)+1\right]$ where *x_c_* is the most probable unbinding force. After performing measurements on two different cell borders and cell surfaces, cells were incubated for 1 h with IgG fractions and mediators and the measurements were repeated on the same areas as before. Finally, incubation induced changes in interaction probability or force were assessed by determination of the ratio of the post-incubation results and the pre-incubation results.

### Triton X-100 Fractionation and Western Blotting

After reaching confluence in 24-well plates, cells were treated with IgG fractions for 24 h. Subsequently, cells were washed twice with ice-cold phosphate-buffered saline (PBS) and lysed in Triton X-100 lysis buffer (0.5 % Triton X-100 (Thermo Fisher Scientific), 50 mM 2-(N-morpholino)ethanesulfonic acid, 25 mM EGTA, 5 mM magnesium chloride, 1 mM phenylmethylsulfonylfluoride, 1 mM aprotinin, 1 mM pepstatin A and 1 mM leupeptin (all from VWR International, Radnor, PA); pH 6.8) on ice for 15 min. Afterwards, cells were scraped from the well bottom and centrifuged for 5 min at 4 °C with 30000x*g*. The resulting pellet containing the cytoskeletal-bound protein fraction was separated from the supernatant comprising the non-cytoskeletal bound molecules. SDS lysis buffer (12.5 mM 4-(2-hydroxyethyl)-1-piperazineethanesulfonic acid (Sigma-Aldrich), 1 mM EDTA (VWR International), 12.5 mM sodium fluoride (VWR International), 0.5 % sodium dodecyl sulfate (Sigma-Aldrich) and a protease inhibitor cocktail according to the manufacturer’s instructions (cOmplete, #11697498001, Roche Diagnostics, Mannheim, Germany); pH 7.4) was used to resuspend the pellet. After sonication the protein concentration was determined using a BCA protein assay kit (Thermo Fisher Scientific) according to the manufacturers protocol. The two lysate fractions were then subjected to Western blot analysis according to standard methods (28). Briefly, lysates were mixed at a ratio of 2:1 with modified Laemmli buffer (29) and subsequently heated to 95 °C for 5 min. Proteins were subjected to electrophoresis and afterwards transferred to nitrocellulose membranes (Thermo Fisher Scientific). Primary antibodies used were anti-GAPDH (#sc-47724, Santa Cruz, Dallas, Tx), anti-Dsg3 (EAP3816, Elabscience, Biozol, Eching, Germany), anti-Dp (A7635, ABclonal, Woburn, MA), anti-Dsg1 (AB9812, ABclonal). Horseradish-peroxidase conjugated secondary antibodies (Dianova, Hamburg, Germany) were used with a self-made enhanced chemiluminescence solution to visualize proteins using an iBright™ FL1500 (Thermo Fisher Scientfic). Quantification was carried out in Image Studio software (LI-COR Biosciences, Lincoln, NE). Band intensities were normalized to GAPDH or Dp for non-cytoskeletal or cytoskeletal fractions, respectively. Resulting values were normalized to the corresponding c-IgG value.

### pSNAPf-hDsg1 Plasmid Generation

The hDsg1 cDNA was amplified by PCR using the mCherry-Desmoglein1-N-18 construct as a template and the primers in **Table 2**. The forward primer, AscI-hDsg1-FW, includes the first 22 bases of the open reading frame of the target of interest with an ATG start codon. In addition, AscI restriction site and Kozak consensus sequence, for enhance translation, are introduced to the 5’ end of the primer. The reverse primer comprises besides the target specific sequence with excluded/omitted STOP codon, an AgeI restriction site. Thus, the resulting PCR product contains an AscI restriction site and Kozak consensus sequence at the 5’ end and an AgeI restriction site at the 3’ end of the target sequence for hDsg1.

**Table 2:** Primer sequences for pSNAPf-hDsg1 generation.

Primer	Sequence 5’➔ 3’
AscI-hDsg1-FW	GGCGCGCCGCCACCATGGACTGGAGTTTCTTCAGAG
AgeI-hDsg1-REV	ACCGGTCTTGCTATATTGCACGGTACTATAC

To improve the efficiency of the restriction digestion of the PCR product, the later was additionally 3’ adenylated and cloned into the pCRTM2.1- TOPO® vector (ThermoFisher Scientific). Afterwards, the target sequence was extracted from the TOPO® vector by digestion with AscI and AgeI restriction enzymes and subcloned into pSNAPf vector (New England Biolabs, N9183S) after linearization with the same restriction enzymes. Thus, the hDsg1 was fused upstream from the SNAP-tag. To ensure that the hDsg1 sequence was correctly introduced into the vector, the resulting plasmid (pSNAPf-hDsg1) was subjected to restriction analyses with either BglII or EcoRI restriction enzymes. The correctness of the construct was further confirmed/verified with sequencing.

### Stimulated Emission Depletion (STED) Microscopy

Cells were seeded on #1.5 coverslips (VWR International). In some experiments cells were transiently transfected with a self-made pSNAPf-hDsg1 plasmid using Turbofect (Thermo Fisher Scientific) according to the manufacturer’s protocol. Confluent cells were incubated with mediators and IgG fractions for 24 h and subsequently fixed with 4 % glyoxal solution (30) for 30 min at room temperature. Washing steps were done using PBS. After permeabilization of cells using 0.1 % Triton X-100 in PBS for 10 min and blocking with 3 % bovine serum albumin and 1 % normal goat serum in PBS primary antibodies anti-Dp (#sc-390975, Santa Cruz) and anti-Dsg1 (AB9812, ABclonal) were applied 1:100 in PBS at 4 °C over night. After washing 3 times with PBS, secondary antibodies conjugated with Alexa Fluor-594 (Abcam, Cambridge, UK) or STAR RED (Abberior, Göttingen, Germany) where incubated 1:200 at room temperature for 1 h. In experiments with hDsg1-SNAP transfected cells SNAP-Cell® TMR-Star (S9195S, New England Biolabs, Ipswitch, MA) was used 1:200 at room temperature to label overexpressed Dsg1. Cells were mounted using ProLong Diamond Antifade Mountant (Thermo Fisher Scientific). STED microscopy was performed on a STED-Expert line setup (Abberior). A 100x 1.4 UPlanSApo objective (Olympus) was used, pixel size was set to 20 nm, a 775 nm pulsed laser at 20 % and a 595 nm pulsed laser at 25 % both with a gating time of 800 ps were used to achieve STED effect. Obtained images were analyzed in areas of “railroad-like” Dp staining using the Coloc2 plugin in ImageJ to obtain the Manders overlap coefficient for the Dsg1 and Dp signal.

### Analysis and Statistics

Statistical analysis was performed using Prism 8 (GraphPad Software, La Jolla, CA). For comparison of two datasets a two-tailed paired Student’s *t*-test was performed. For more than two datasets two-way ANOVA corrected by Holm-Sidak’s *Post-hoc* test was used to determine significance. Significance was assumed for *P<0.05. Error bars represent standard deviation. Images and figures were arranged using Photoshop and Illustrator (Adobe, San José, CA). Micrograph analyzation was done using Image J software (National Institutes of Health).

## Results

### Suppression of Ca^2+^ and ERK1/2 Signaling Ameliorates Loss of PF-IgG-Induced Intercellular Adhesion

**Figure 1:**
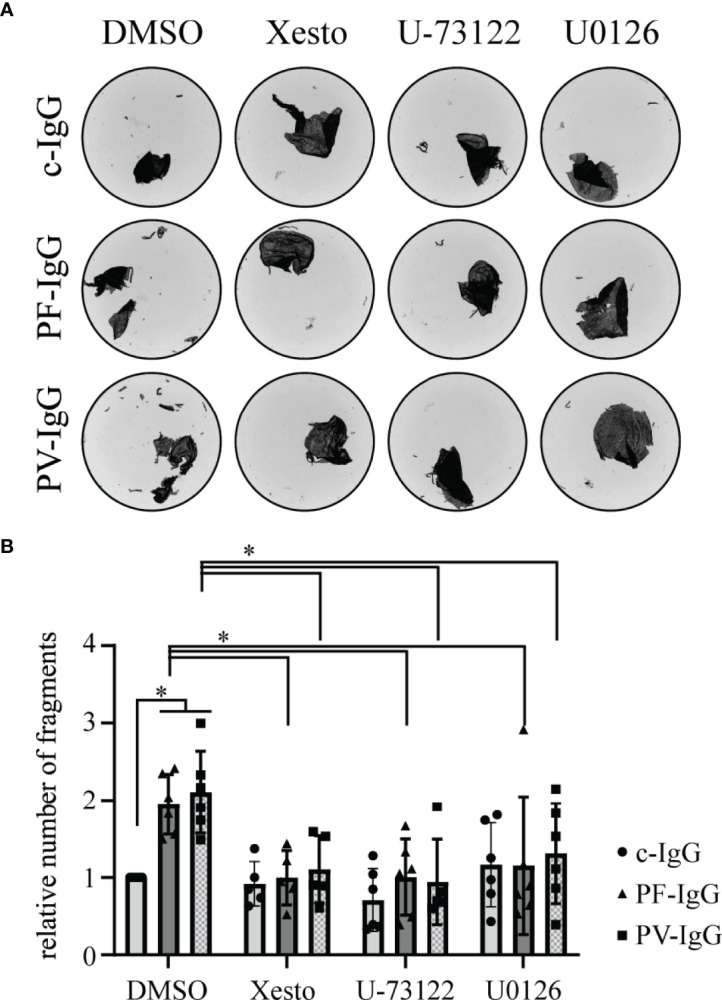
Pemphigus autoantibody induced loss of cell cohesion can be ameliorated by inhibition of Ca^2+^ and ERK1/2 signaling; **(A)** Representative images of monolayer fragments after the application of shear stress of dispase-based dissociation assays of HaCaT cells after 24 h incubation with IgG fractions and mediators. **(B)** Corresponding quantification to **(A)**; the number of fragments in each experimental condition has been normalized to the DMSO c-IgG value. N ≥ 5; each dot represents one independent experiment. *P < 0.05 in two-way ANOVA; error bars represent standard deviation.

Desmosomal adhesion has long been shown to be regulated by a variety of signaling pathways and recent studies suggest involvement of Ca^2+^ signaling in pemphigus pathogenesis (19, 22, 23). In this study, we investigated the effects of IgG fractions from sera from PF patients *in vitro* in HaCaT cells with respect to possible pathomechanisms. In dispase-based dissociation assays, the loss of intercellular adhesion, which is typical for treatment with pemphigus patients’ IgG fractions, was confirmed (31, 32) (**Figures 1A, B**). Fully confluent HaCaT cells were treated for 24 h with IgG fractions before being subjected to dissociation assays. Pathogenic PF-IgG and PV-IgG led to a significant increase in fragment numbers compared to incubation with c-IgG from a healthy control donor. In normal human epidermal keratinocytes, it could be shown that inhibition of Ca^2+^ and ERK1/2 signaling was sufficient to suppress loss of intercellular adhesion in dissociation assays (23). As a next step, we investigated if these effects can also be observed in HaCaT cells. Ca^2+^ influx was blocked either by inhibition of IP3R activation *via* xestospongin C (Xesto) or further upstream by blocking IP3 synthesis *via* PLC inhibition using U-73122. U0126 was used to inhibit ERK1/2 signaling *via* MEK1/2 activity suppression which is the upstream kinase of ERK1/2. Incubation with mediators started 1 h prior to 24 h IgG treatment and resulted in suppression of fragmentation in response to mechanical stress for pathologic IgG fractions (**Figures 1A, B**; **Supplementary Figures 1A, B**). It is worth noting, that overexpression of Dsg1 has no protective effect against loss of cell adhesion induced by PF-IgG (**Supplementary Figure 2A, B**).

These data demonstrate that pathogenic PF-IgG used in this study were effective to induce loss of adhesion in keratinocytes, a hallmark for pemphigus pathogenicity, similar to PV-IgG containing both antibodies against Dsg1 and Dsg3.

### PF-IgG Reduce Dsg1 Binding Probability But Not Single-Molecule Binding Force in Atomic Force Measurements

**Figure 2:**
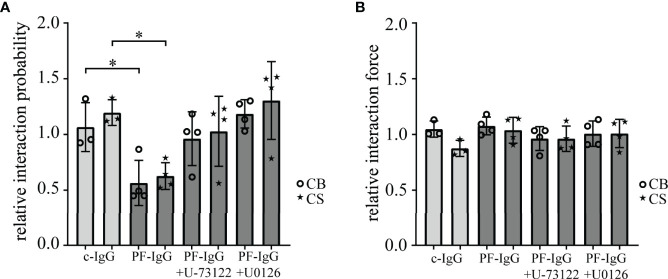
AFM based single-molecule force spectroscopy reveals reduced Dsg1 interaction upon PF-IgG incubation which can be rescued by blocking of ERK1/2 or PLC signaling. Interaction probability **(A)** and force **(B)** of desmoglein 1 coated AFM cantilevers after 1 h incubation with IgG and selected mediators compared to pre-incubation values. Measurements were either conducted across the border of two cells (CB) or on the surface above the nucleus of one cell (CS). N ≥ 3; each dot represents the mean value of ≥800 force‒distance curves from one independent experiment. *P < 0.05 in two-way ANOVA; error bars represent standard deviation.

To investigate whether decreased intercellular adhesion in response to PF-IgG correlates with alterations of Dsg1 binding properties on the single molecule level, atomic force microscopy (AFM) was used. Cantilevers functionalized with the complete recombinant Dsg1 extracellular domain linked to human Fc were used to probe living HaCaT cells as described previously (4). Measurements were conducted as well in an area spanning the cell border (CB) between two cells as on top of a single cell in an area above the nucleus (CS). After measuring single molecule interaction forces and probabilities under baseline conditions, cells were incubated for 1 h with IgG fractions and mediators. Then, measurements were repeated and resulting changes in interaction forces and probabilities were calculated (**Figures 2A, B**). During all conditions no significant differences in binding force or binding probability between cell border and cell surface areas could be detected. Likewise, the interaction forces did not change in response to c-IgG, PF-IgG alone or in addition with U-73122 or U0126. Regarding the interaction probability, incubation with c-IgG did not lead to alterations in quantitative binding behavior. PF-IgG incubation on the other hand resulted in a substantial reduction in binding probability, roughly halving the number of binding events after PF-IgG incubation. This effect was abolished when PF-IgG was co-incubated with U-73122 or U0126, respectively.

Taken together, these data demonstrate that in response to PF-IgG Dsg1 molecules become rapidly less available for single molecule measurements, but the interaction force of individual binding events is not altered. This effect can be blocked by inhibiting either Ca^2+^ or ERK1/2 signaling. Thus, effects of PF-IgG and involved signaling on loss of keratinocyte adhesion is paralleled by loss of Dsg1 binding on the molecular level, in line with the interpretation that Dsg1 adhesion is disturbed by autoantibodies.

### PF-IgG Reduce Dsg1 Mobility in the Cell Membrane

**Figure 3:**
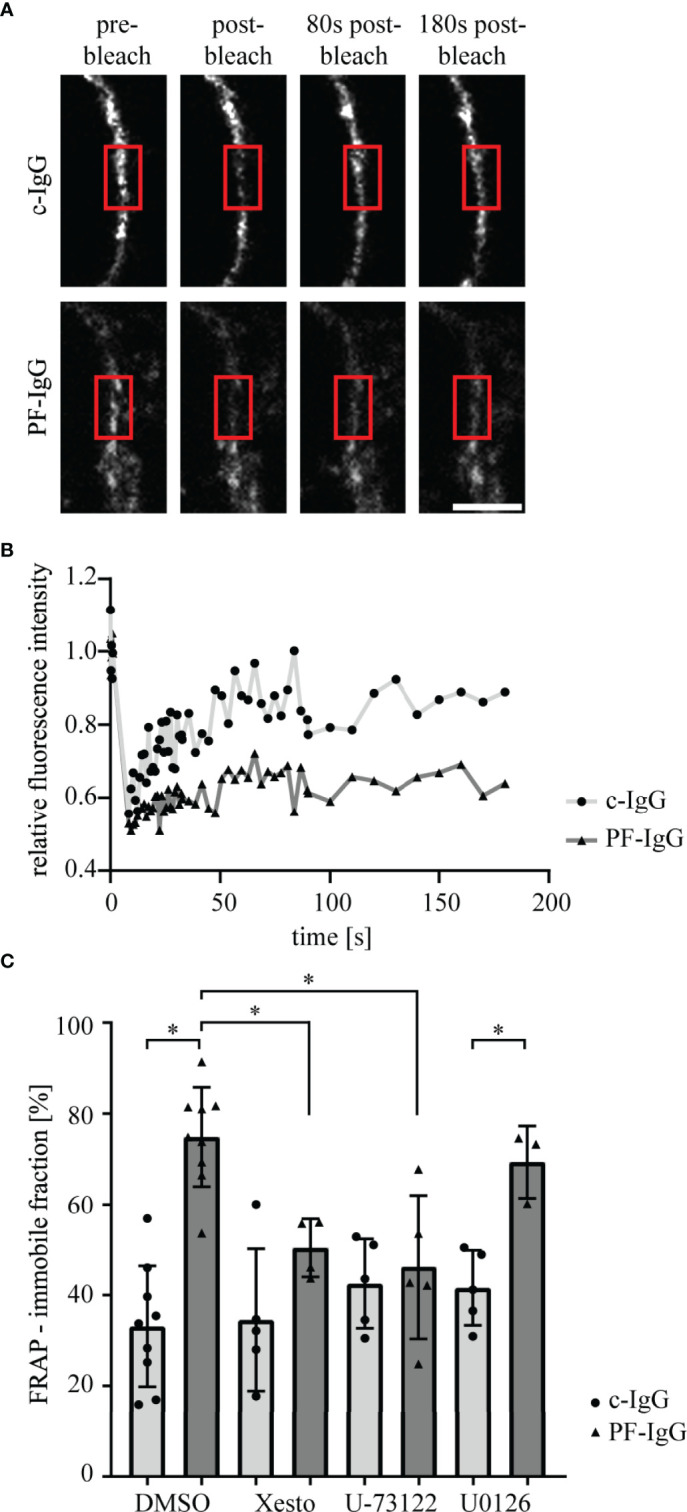
Fluorescence recovery after photobleaching of Dsg1-mCherry is impaired after incubation with PF-IgG but can be rescued by blocking of PLC or Ca^2+^ signaling. **(A)** Representative images of fluorescence recovery after photobleaching (FRAP) time series. Cells were incubated for 24 h with IgG fractions and mediators. A part of the cell membrane between two neighboring cells is recorded, then the area marked with the red box is bleached with the laser as apparent by the drop in fluorescent intensity and the recovery of the fluorescence is monitored over 3 minutes. Scale bar: 5µm. **(B)** Corresponding fluorescence intensity recovery curves to **(A)**; normalized to pre-bleach intensity. **(C)** Quantification of the average fraction of immobile Dsg1-mCherry molecules as calculated from fits to fluorescence recovery curves. N ≥ 3; each dot represents the mean of ≥3 FRAP experiments in ≥3 different cell pairs of one independent experiment. *P < 0.05 in two-way ANOVA; error bars represent standard deviation.

To investigate underlying mechanisms for loss of intercellular adhesion after PF-IgG incubation, we analyzed Dsg1 dynamics using FRAP (33). Cells were transiently transfected with Dsg1-mCherry and treated for 24 h with mediators and IgG fractions. After bleaching of Dsg1-mCherry signal at the cell border between two transfected cells the immobile fraction of molecules was determined. Incubation with PF-IgG drastically increased the pool of immobile Dsg1-mCherry molecules in the cell membrane (**Figures 3A–C**). Incubation with signaling pathway inhibitors led to no change in immobile fraction when treating the cells with c-IgG. However, the inhibitors Xesto and U-73122 but not U0126 were effective to abolish the increase in immobile fraction in response to PF-IgG. Taken together, these results indicate involvement of Ca^2+^ but not ERK1/2 signaling in modulation of membrane bound Dsg1 mobility.

### PF-IgG Lead to Redistribution of Membrane-Bound Dsg1

**Figure 4:**
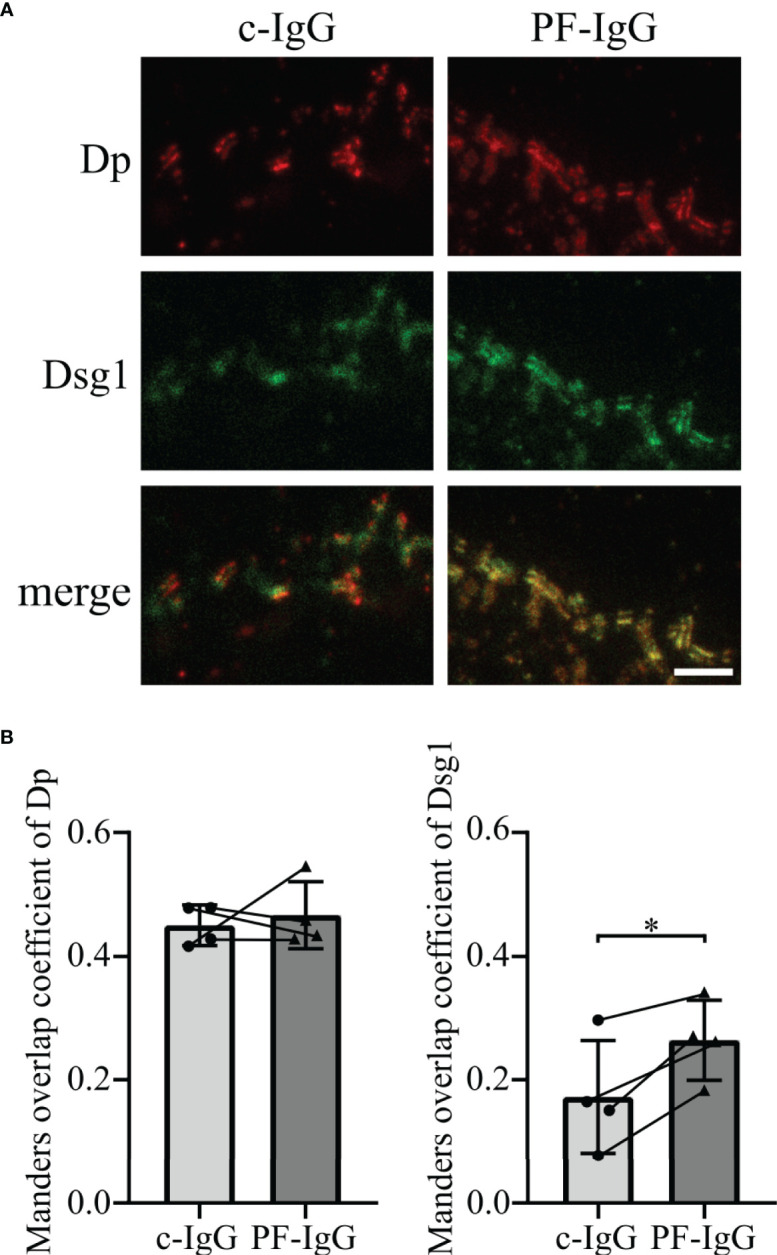
Incubation of PF-IgG leads to redistribution of membrane-bound, overexpressed Dsg1 in HaCaT cells. **(A)** Representative immunostainings of HaCaT cells overexpressing desmoglein 1 (Dsg1) and incubated 24 h with IgG fractions, stained for desmoplakin (Dp) and overexpressed Dsg1. Scale bar: 1 µm. **(B)** Manders overlap coefficient of Dp with Dsg1 (left) and Dsg1 with Dp (right). N=4; each dot represents the mean of >5 acquisitions from one independent experiment. *P<0.05 in two-tailed paired Student’s t-test; error bars represent standard deviation.

To further explore the fate of Dsg1 in response to PF-IgG, STED microscopy was performed to optically differentiate between desmosomal and extra-desmosomal Dsg1. To this end, cells were transiently transfected to overexpress Dsg1 to correlate the results with the findings of Dsg1 overexpression in FRAP experiments. The high spatial resolution of STED images allowed us to use Dp immunostaining to identify single desmosomes, clearly recognizable due to the “railroad track”-like appearance Dp exhibits when localized in desmosomes (34). Cells treated with c-IgG for 24 h showed regularly formed desmosomes and Dsg1 staining was colocalized with Dp, indicating desmosome-bound Dsg1, as well as outside the desmosomes along the cell membrane (**Figure 4A**). Incubation with PF-IgG on the other hand increased colocalization of Dsg1 with Dp with apparently less extradesmosomal Dsg1. Evaluation of the Manders overlap coefficient (35) along cell borders showing desmosomes resulted in an unchanged probability for Dp signal to be colocalized with Dsg1 signal in response to PF-IgG incubation (**Figure 4B** left). However, PF-IgG treatment led to an increased probability of Dsg1 signal to overlap with Dp signal (**Figure 4B** right).

These results imply that PF-IgG incubation results in a redistribution of membrane-bound Dsg1 rather than of Dp.

### PF-IgG Induced Redistribution of Dsg1 Is Dependent on Ca^2+^ Signaling

**Figure 5:**
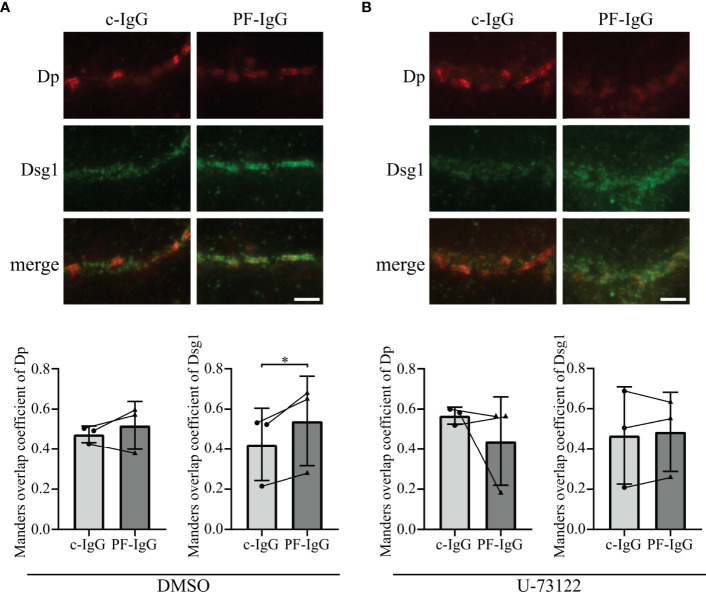
PF-IgG induced redistribution of Dsg1 within the cell membrane can be prevented by PLC inhibition. **(A)** Top: Representative immunostainings of HaCaT cells stained for desmoglein 1 (Dsg1) and desmoplakin (Dp), incubated 24 h with IgG fractions and DMSO as control. Scale bar: 1 µm. Bottom: Manders overlap coefficient of Dp with Dsg1 (left) and Dsg1 with Dp (right). N=3; each dot represents the mean of >3 acquisitions from one independent experiment. *P<0.05; error bars represent standard deviation (SD). **(B)** Top: Representative immunostainings of HaCaT cells stained for Dsg1 and Dp, incubated 24 h with IgG fractions and U-73122. Scale bar: 1 µm. Bottom: Manders overlap coefficient of Dp with Dsg1 (left) and Dsg1 with Dp (right). N=3; each dot represents the mean of >3 acquisitions from one independent experiment. *P<0.05 in two-tailed paired Student’s t-test; error bars represent standard deviation.

Thereafter, we assessed if endogenous Dsg1 behaves comparable to over expressed Dsg1 in this regard. After reaching confluency, cells were treated with IgG fractions for 24 h and stained for Dsg1 and Dp (**Figure 5A**). STED microscopy revealed the same Dsg1 distribution pattern as described above for overexpression of Dsg1. Dsg1 can be found ubiquitous along the cell membrane if treated with c-IgG but shows significantly enhanced colocalization with Dp after PF-IgG incubation. Co-incubation of PLC inhibitor U-73122 with IgG fractions abolished the effects of PF-IgG on Dsg1 relocalization leading to more uniformly distributed Dsg1 signal along the cell membrane (**Figure 5B**). Taken together, these findings support the notion that Ca^2+^ signaling plays a crucial role in cellular PF-IgG response.

**Figure 6:**
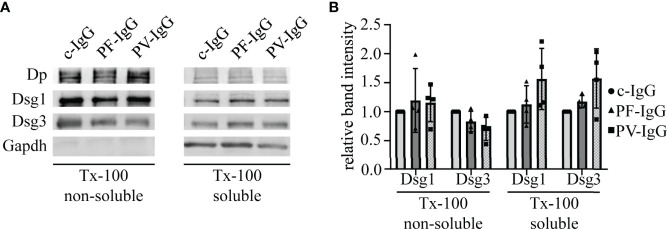
Pemphigus IgG fractions do not induce changes in the distribution of Dsg1 and Dsg3 with respect to cytoskeletal anchorage. **(A)** Representative Western blot of Triton X-100 (Tx-100) fractionations of HaCaT cells incubated for 24 h with IgG fractions. **(B)** Corresponding quantification to **(A)**. Band intensities were normalized to GAPDH or desmoplakin (Dp) for non-cytoskeletal or cytoskeletal fractions, respectively. Resulting values were normalized to the corresponding c-IgG value. N=4; error bars represent standard deviation.

Because the data conclusively demonstrate that PF-IgG within the first 24 h predominantly caused a redistribution of extradesmosomal Dsg1 and it was shown that depletion of Dsg molecules under some experimental conditions can be studied using protein fractionation (36), we studied whether PF-IgG resulted in an uncoupling of Dsgs from the cytoskeleton. Therefore, we performed Triton X-100 fractionations and visualized protein fractions with Western blot analyses (**Figures 6A, B**). Neither PF-IgG nor PV-IgG incubation for 24 h showed significant alterations of Dsg1 or Dsg3 localization in Triton X-100 soluble or non-soluble lysate fractions compared to c-IgG treatment. These results indicate that the proteins amounts of Dsg1 and Dsg3 localized in both the cytoskeleton-bound desmosomal fraction and the non-cytoskeleton-bound extradesmosomal fraction do not change significantly during 24 h of autoantibody incubation.

## Discussion

Pemphigus is a rare disease and its treatment currently is mainly focused on systemic immunosuppression (37). More specific treatments are urgently needed and some compounds seem to be promising candidates, including an inhibitor of MEK1/2 which is upstream of ERK1/2 signaling (38). Since the exact pathomechanism in pemphigus remains elusive, we attempted to further investigate the role of Dsg1, as antibodies directed against Dsg1 appear crucial for skin blistering in pemphigus (39). In this study, we employed a combination of powerful biophysical techniques such as life cell AFM, FRAP and super resolution STED microscopy and identified PLC and therefore Ca^2+^ signaling to play a crucial role in relocalization of extradesmosomal Dsg1 in response to PF-IgG. PLC signaling has long ago been shown to be involved in pemphigus IgG-induced signaling in cultured keratinocytes and most recently was demonstrated to contribute to acantholysis and keratin uncoupling from desmosomes in human skin (23, 40, 41).

Our data confirmed pathogenicity of the employed IgG fractions using dispase-based dissociation assays (23, 31, 32, 42). By suppression of Ca^2+^ signaling *via* PLC and IP3 receptor inhibition we were able to rescue intercellular adhesion in response to pemphigus IgG. Similarly, inhibition of ERK1/2 was sufficient to maintain cell cohesion after exposure to pathogenic IgG. These results are in good accordance with previously published studies (42). Because changes in keratinocyte adhesion were paralleled by alterations in Dsg1 binding frequency on the molecular level as revealed by AFM, these experiments indicate that PF-IgG presumably reduce cell adhesion by targeting Dsg1 in a manner dependent on both PLC/Ca^2+^ and ERK1/2. Thereafter, we investigated the dynamics of Dsg1 in the cell membrane using FRAP. We observed a striking decrease in Dsg1 mobility after PF-IgG treatment. One reason for this, which would also be compatible with the results obtained by AFM, might be an internalization of Dsg1 molecules, as often observed (43, 44). This would result in the remaining Dsg1 proteins being localized to the desmosomes and thus strongly restricted in their movement. These effects could be ameliorated by inhibition of PLC or IP3 receptor but interestingly not *via* inhibition of ERK1/2 even though it was sufficient to prevent cell sheet fragmentation in dissociation assays and loss of Dsg1 binding as detected by AFM. One may conclude that desmosome adhesion is regulated by several pathways including PLC/Ca^2+^ and ERK1/2 whereas relocalization of extradesmosomal Dsg1 after PF-IgG treatment is ERK1/2-independent. This is interesting because it was suggested that antibodies against Dsg1 activate both pathways by using Dsg1 as adhesion-dependent signaling receptor (31). Indeed, PLC and its upstream kinase PI4-kinase have been found to be associated with Dsg1 (23).

It has been shown by AFM that PF-IgG lead to a redistribution of Dsg1 molecules from cell borders to the cell surface, at least in murine keratinocytes, in response to PF-IgG (45). However, we did not find similar redistribution of Dsg1. These discrepancies could be explained either by the different cell species or by differences in PF-IgG composition since patient’s sera may contain a multitude of different antibodies which might contribute to pathogenic effects (46) as well as it has been shown that depending on the stage of the disease the autoantibodies recognize different epitopes of Dsg1 (47). The same holds true for other studies which described a reduction of Dsg1 in the Triton X-100 soluble pool, i.e., non-cytoskeletal anchored Dsg1, an effect we were not able to observe (44). It is likely that this difference, which previously also was noted for Dsg3 in fully confluent HaCaT cells compared to subconfluent keratinocytes (36, 48), is due to different maturation states of desmosomes as has been shown in a study comparing different culture conditions and time points after Ca^2+^-induced desmosome formation (49). Using super resolution microscopy, we confirmed our hypothesis derived from FRAP experiments that Dsg1 remains in desmosomes and during the first 24 h mainly is depleted from extradesmosomal sites. This was validated for both endogenous and overexpressed Dsg1 levels. In both cases PF-IgG led to a clearly visible confinement of Dsg1 to desmosomes whereas it was ubiquitously distributed along the membrane under control conditions. Consistent with FRAP results inhibition of PLC abolished this effect, indicating that PLC is involved in mediating altered Dsg1 dynamics and localization. These results obtained by STED support the observation from Triton X-100 mediated protein extraction that Dsg1 in the cytoskeletal-bound fraction, which comprises the majority of Dsg1, was not altered. However, the relatively small amount of Dsg1 in the cytoskeletal-unbound fraction was also not altered by incubation with PF-IgG although FRAP and STED experiments indicated depletion of extradesmosomal Dsg1. The same has been described in cardiomyocytes under conditions of enhanced intercellular adhesion where Dsg2 translocates to cell junctions together with Pg and Dp (50, 51). An explanation would be, that during final steps of desmosome formation and maturation Dsg molecules coupled to Dp and intermediate filaments become trapped in desmosomes whereas the cytoskeletal anchorage is not changed which has been described recently for hyper-adhesion (52). Alternatively, it has to be noted that the Triton X-100-soluble Dsg1 pool contains also molecules removed from the cell membrane into cytoplasmic vesicles which also would account for a reduced mobile fraction in FRAP.

Altogether, these data support a crucial role of PLC/Ca^2+^ and ERK1/2 signaling for regulation of Dsg1-mediated binding in PF pathogenesis and suggest that PLC/Ca^2+^ besides adhesion also controls extradesmosomal Dsg1 localization. If it is true that extradesmosomal Dsg contacts serve as signaling molecules (19) it can be concluded that autoantibodies in pemphigus during the first 24 h after binding cause signaling and interfere with assembly of new desmosomes before intact desmosomes become disassembled or down-regulated (53).

## Data Availability Statement

The raw data supporting the conclusions of this article will be made available by the authors, without undue reservation.

## Ethics Statement

Patients gave written informed consent for use of their blood sera in research. It was reviewed and approved by the Ethics Committee of the University of Marburg (Az20/14).

## Author Contributions

MHi conducted the experiments, acquired and analyzed the data. MHi, DK and MR developed the methodology. DD, KG, SF and MHe contributed patients’ sera. MHi and JW interpreted the data, designed the research study and wrote the manuscript. All authors contributed to the article and approved the submitted version

## Funding

This work was supported by DFG FOR 2497 TP5 to JW.

## Conflict of Interest

The authors declare that the research was conducted in the absence of any commercial or financial relationships that could be construed as a potential conflict of interest.

## Publisher’s Note

All claims expressed in this article are solely those of the authors and do not necessarily represent those of their affiliated organizations, or those of the publisher, the editors and the reviewers. Any product that may be evaluated in this article, or claim that may be made by its manufacturer, is not guaranteed or endorsed by the publisher.

## Supplementary Material

Supplementary Figure 1 AK23 shows similar decrease in intercellular adhesion as PF-IgG and PV-IgG in dispase-based dissociation assays. **(A)** Absolute number of fragments corresponding to **Figure 1B**. **(B)** Top: Quantification of dispase-based dissociation assays on HaCaT cells after 24 h incubation with IgG fractions and AK23. N=4; each dot represents one independent experiment. *P < 0.05 in one-way ANOVA vs. c-IgG; error bars represent standard deviation. Bottom: Representative images of monolayer fragments.

Supplementary Figure 2 Overexpression of Dsg1 does not alter intercellular adhesion in dispase-based dissociation assays. **(A)** Representative images of monolayer fragments after the application of shear stress of dispase-based dissociation assays of HaCaT cells 48 h after transfection and after 24 h incubation with c-IgG or PF-IgG, respectively. Cells have either been transfected with no plasmid (Mock), pSNAPf-hDsg1 (SNAP) or mCherry-Desmoglein1-N-18 (Cherry), respectively. **(B)** Corresponding quantification to **(A)**; Left: The number of fragments in each experimental condition has been normalized to the c-IgG Mock value. Right: Absolute number of fragments; N=3; each dot represents one independent experiment. *P < 0.05 in two-way ANOVA; error bars represent standard deviation.
