# Supplementary figures and images for: Pemphigus Foliaceus Autoantibodies Induce Redistribution Primarily of Extradesmosomal Desmoglein 1 in the Cell Membrane
Source: Front Immunol. 2022 May 12;13:882116. doi: 10.3389/fimmu.2022.882116 (PMC9134081; doi:10.3389/fimmu.2022.882116)

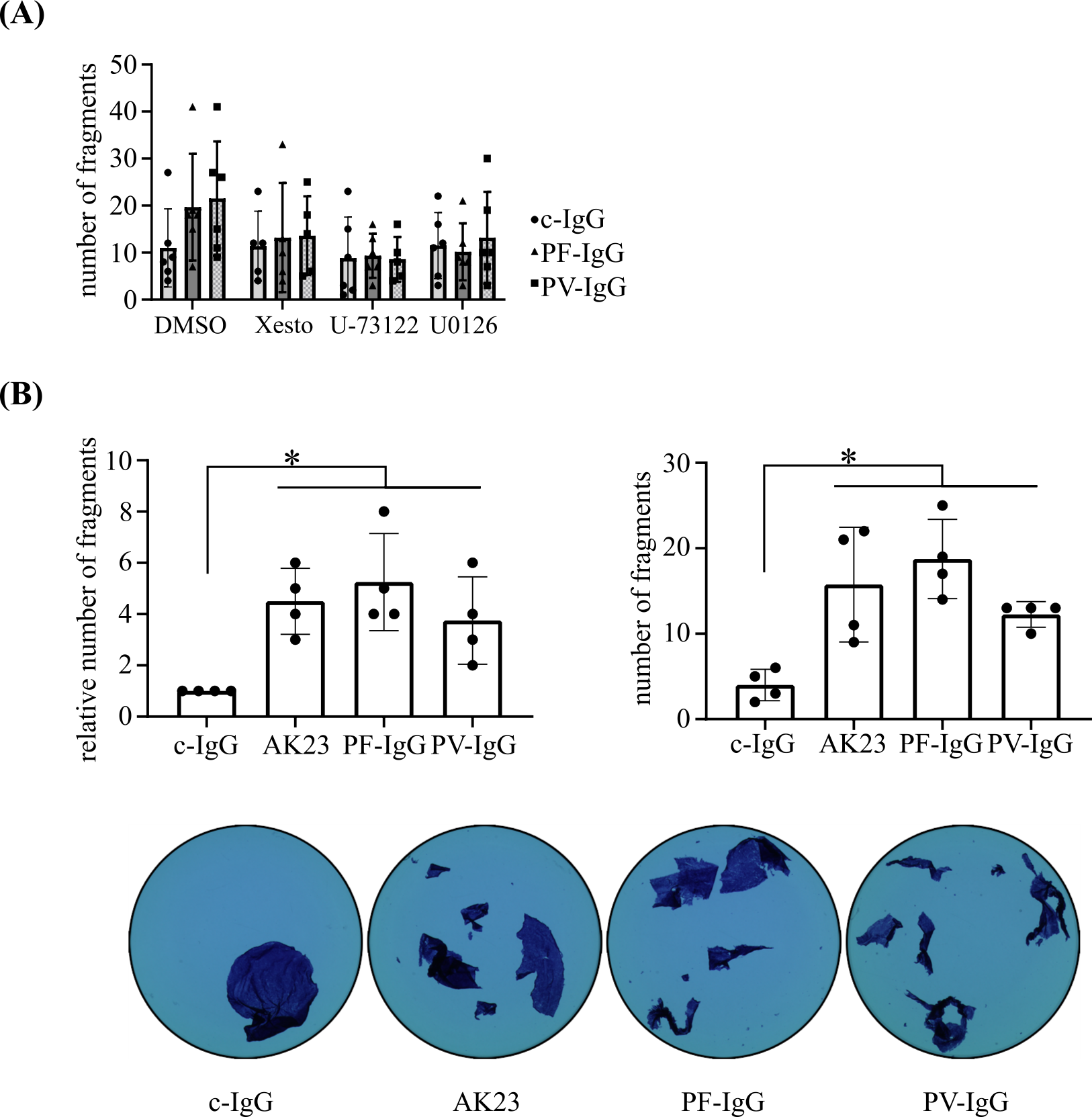

Supplement: Supplementary Figure 1 — AK23 shows similar decrease in intercellular adhesion as PF-IgG and PV-IgG in dispase-based dissociation assays. (A) Absolute number of fragments corresponding to Figure 1B. (B) Top: Quantification of dispase-based dissociation assays on HaCaT cells after 24 h incubation with IgG fractions and AK23. N=4; each dot represents one independent experiment. *P < 0.05 in one-way ANOVA vs. c-IgG; error bars represent standard deviation. Bottom: Representative images of monolayer fragments. [file Image_1.tif]

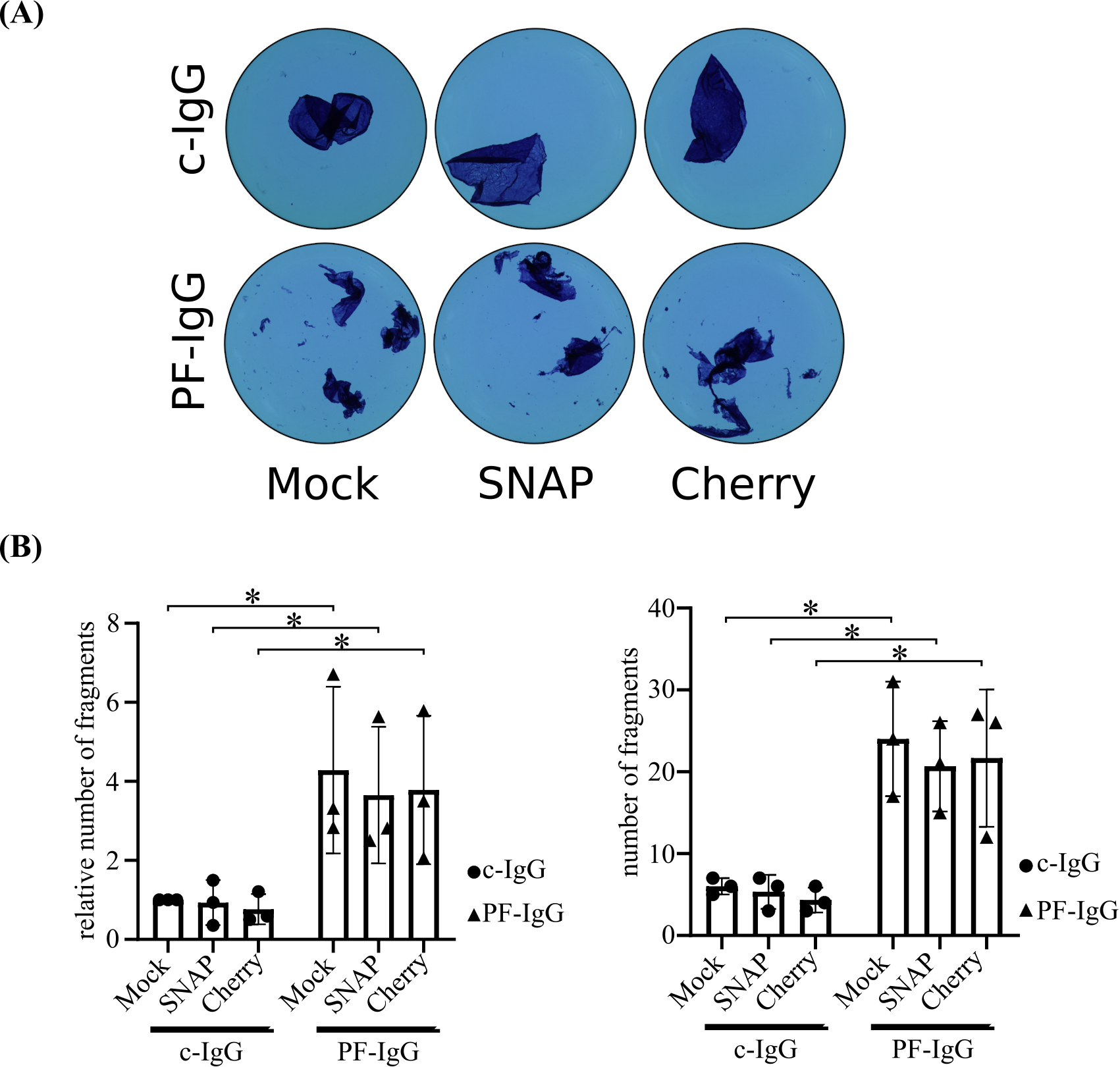

Supplement: Supplementary Figure 2 — Overexpression of Dsg1 does not alter intercellular adhesion in dispase-based dissociation assays. (A) Representative images of monolayer fragments after the application of shear stress of dispase-based dissociation assays of HaCaT cells 48 h after transfection and after 24 h incubation with c-IgG or PF-IgG, respectively. Cells have either been transfected with no plasmid (Mock), pSNAPf-hDsg1 (SNAP) or mCherry-Desmoglein1-N-18 (Cherry), respectively. (B) Corresponding quantification to (A); Left: The number of fragments in each experimental condition has been normalized to the c-IgG Mock value. Right: Absolute number of fragments; N=3; each dot represents one independent experiment. *P < 0.05 in two-way ANOVA; error bars represent standard deviation. [file Image_2.tif]
